# Independent associations of physical activity and depression with open-angle glaucoma in a population-based analysis

**DOI:** 10.1038/s41598-025-28364-0

**Published:** 2025-11-21

**Authors:** Sang Yeop Lee, Sang-Jun Shin, Jeongwoo Lee, David Myung, Hun Lee

**Affiliations:** 1 Department of Ophthalmology, Yongin Severance Hospital, Yonsei University College of Medicine, Yongin, Korea; 2 Institute of Vision Research, Department of Ophthalmology, Yonsei University College of Medicine, Seoul, Korea; 3 Data Research, Samil Pharm Co. LTD, Seoul, Korea; 4 Department of Ophthalmology, Spencer Center for Vision Research, Byers Eye Institute at Stanford University, Palo Alto, CA USA; 5 Department of Ophthalmology, Asan Medical Center, University of Ulsan College of Medicine, 88, Olympic-Ro 43-Gil, Songpa-Gu, Seoul, 05505 South Korea; 6 Department of Ophthalmology, Brain Korea 21 project, University of Ulsan College of Medicine, Seoul, Korea; 7 Center for Cell Therapy, Asan Medical Center, Seoul, Korea

**Keywords:** Open angle glaucoma, Depression, Physical activity, Korean national health insurance service, Diseases, Health care, Medical research, Risk factors

## Abstract

Patients with open-angle glaucoma (OAG), a chronic visual impairment, are at elevated risk of developing depression. Physical activity (PA) has been associated with benefits for mental health, but evidence is limited regarding its impact on depression risk in individuals with OAG. This study aimed to assess the association between PA and incident depression in a nationwide cohort of patients newly diagnosed with OAG. We conducted a retrospective cohort study using customized data from the Korean National Health Insurance Service (KNHIS). The cohort included 97,617 individuals newly diagnosed with OAG between 2011 and 2015, with follow-up until depression onset, death, or study end in 2023. Age- and sex-matched controls without OAG were selected at a 1:2 ratio. PA levels were classified as low or high, based on validated self-report, and categorized into four patterns according to change before and after OAG diagnosis: consistently low, consistently high, increased (low to high), and decreased (high to low). The primary outcome was incident depression identified using ICD-10 diagnostic codes. Multivariable Cox proportional hazards regression was used to estimate hazard ratios (HRs) and 95% confidence intervals (CIs) for depression risk according to PA patterns. Among patients with OAG (mean [SD] age, 59.7 [12.5] years; 42.5% female), those maintaining high PA levels after diagnosis had a lower risk of developing depression compared with those with low PA (HR, 0.877; 95% CI, 0.856–0.900). The most favorable outcomes was observed in patients with consistently high PA (HR, 0.855; 95% CI, 0.829–0.883), followed by those who increased their PA post-diagnosis (HR, 0.912; 95% CI, 0.879–0.947). Patients who decreased PA or remained inactive showed no significant reduction in depression risk. Overall, Higher or increasing physical activity after OAG diagnosis was associated with a modest but meaningful reduction in depression risk. These findings suggest that encouraging PA may help mitigate depression risk and should be considered as part of comprehensive glaucoma care.

**Supplementary Information:**

The online version contains supplementary material available at 10.1038/s41598-025-28364-0.

## Introduction

Glaucoma is a progressive optic neuropathy characterized by functional impairment of the optic nerve, and is a leading cause of blindness worldwide. It presents a significant burden on patients and healthcare systems. The chronic, lifelong nature of glaucoma, along with the potential for vision loss, often imposes a psychological burden on patients, which significantly affects their quality of life^1^. Studies have consistently shown that individuals with chronic diseases are at increased risk of developing psychiatric disorders, including depression^2–4^. For instance, research has demonstrated a higher prevalence of depression among patients with diabetes and cardiovascular disease^5–7^. Similarly, several studies have highlighted an association between glaucoma and depression^8–10^. Recent evidence has emphasized the role of physical activity (PA) in mitigating the risk of depression across various chronic illnesses^11–13^. This effect is attributed to its positive impact on neuroplasticity, stress reduction, and systemic inflammation^14,15^. Importantly, glaucoma is a lifelong disease, and longitudinal perspectives are crucial to understanding how changes in patient behaviors influence outcomes. In this context, examining PA before and after the diagnosis of OAG provides insight into whether maintaining or modifying PA trajectories influences the risk of depression. Recent studies of other chronic diseases, including cardiovascular disease, diabetes, and cancer, have similarly investigated PA changes before and after diagnosis to evaluate how behavioral adaptations shape long-term prognosis^16–20^. However, to our knowledge, no study has explored this question in patients with glaucoma.

Therefore, this study aimed to investigate the role of PA in the risk of developing depression among patients with open angle glaucoma (OAG). We utilized the customized database of the Korean National Health Insurance Service (KNHIS) to determine the impact of PA before and after a diagnosis of OAG on the subsequent development of depression.

## Results

### Baseline characteristics

Overall, 97,617 patients diagnosed with OAG participated in this study. These patients were categorized into two groups: those who developed depression after their OAG diagnosis (*n* = 25825) and those who did not (*n* = 71792). The average time from OAG diagnosis to the onset of depression was 3.98 ± 2.71 years. A comparison of baseline characteristics between the two groups revealed significant differences in all variables, except for Body mass index (BMI) and PA level pre-OAG diagnosis (Table 1). Patients in the depression group were older (64.0 ± 11.3 vs. 58.2 ± 12.6 years, *p* < 0.001) and comprised a higher proportion of females (52.4% vs. 40.3%, *p* < 0.001) than those in the no-depression group. The prevalence rates of comorbidities, including hypertension (53.6% vs. 43.9%, *p* < 0.001), diabetes (23.4% vs. 18.9%, *p* < 0.001), and dyslipidemia (40.7% vs. 34.7%, *p* < 0.001) were significantly higher in the depression group than in the no-depression group. Similarly, the Charlson Comorbidity Index (CCI) was higher in the depression group (2.6 ± 2.1 vs. 1.9 ± 1.9, *p* < 0.001), with a greater proportion of patients having CCI ≥ 3 (44.8% vs. 29.4%, *p* < 0.001). While PA levels pre-OAG diagnosis were not significantly different between the groups, PA levels post-OAG diagnosis showed a minimal but significant difference (*p* = 0.001).

**Table 1:** Baseline characteristics of open angle glaucoma patients stratified by depression status.

	Non-depression	Depression	*P*-value
	(*N* = 71792)	(*N* = 25825)	
Age (year, N)	58.2 ± 12.6	64.0 ± 11.3	< 0.001
< 40	5982 ( 8.3%)	795 ( 3.1%)	< 0.001
40–49	11,874 (16.5%)	2150 ( 8.3%)	
50–59	18,705 (26.1%)	4993 (19.3%)	
60–69	20,452 (28.5%)	8568 (33.2%)	
70–79	12,954 (18.0%)	8149 (31.6%)	
≥ 80	1825 ( 2.5%)	1170 ( 4.5%)	
Sex			< 0.001
Male	42,834 (59.7%)	12,297 (47.6%)	
Female	28,958 (40.3%)	13,528 (52.4%)	
Comorbidities			
Hypertension	31,483 (43.9%)	13,838 (53.6%)	< 0.001
Diabetes	13,581 (18.9%)	6044 (23.4%)	< 0.001
Dyslipidemia	24,877 (34.7%)	10,516 (40.7%)	< 0.001
CCI	1.9 ± 1.9	2.6 ± 2.1	< 0.001
0	18,432 (25.7%)	3655 (14.2%)	< 0.001
1	18,822 (26.2%)	5267 (20.4%)	
2	13,413 (18.7%)	5343 (20.7%)	
≥ 3	21,125 (29.4%)	11,560 (44.8%)	
BMI (kg/m^2^)	24.1 ± 3.1	24.0 ± 3.1	0.082
Alcohol consumption			< 0.001
Non	40,956 (57.0%)	17,641 (68.3%)	
Mild	18,913 (26.3%)	5035 (19.5%)	
Moderate	7055 ( 9.8%)	1753 ( 6.8%)	
Severe	4868 ( 6.8%)	1396 ( 5.4%)	
Smoking			< 0.001
Never smoker	43,289 (60.3%)	17,758 (68.8%)	
Ex-smoker	16,111 (22.4%)	4683 (18.1%)	
Current smoker	12,392 (17.3%)	3384 (13.1%)	
Low income			< 0.001
The bottom 20% or below	9854 (13.7%)	4087 (15.8%)	
Others	61,938 (86.3%)	21,738 (84.2%)	
Physical activity level			
Before OAG diagnosis			0.065
Low level	32,363 (45.1%)	11,469 (44.4%)	
High level	39,429 (54.9%)	14,356 (55.6%)	
After OAG diagnosis			0.001
Low level	30,936 (43.1%)	11,452 (44.3%)	
High level	40,856 (56.9%)	14,373 (55.7%)	

Data are expressed as the mean ± the standard deviation or as n (%). The P-value is derived from the student t-test and the Chi-square test. BMI, body mass index; CCI. Charlson comorbidity index; OAG. open angle glaucoma. Patients who developed depression after OAG diagnosis were older, more often female, and had a higher prevalence of comorbidities compared with those without depression.

### Comparison between groups based on PA levels

Table 1 presents significant group differences in PA levels post-OAG diagnosis. Therefore, we compared the two groups stratified by PA levels to further investigate potential associations. All variables, excluding BMI, exhibited significant differences between the two groups stratified by PA level post-OAG diagnosis (Table 2). The prevalence of depression was significantly lower in the high PA level group than in the low PA group (*p* = 0.001).

**Table 2:** Comparison based on physical activity level after open angle glaucoma diagnosis.

	PA low level	PA high level	*P*-value
	(*N* = 42388)	(*N* = 55229)	
Age (year, N)	57.9 ± 12.4	61.2 ± 12.4	< 0.001
< 40	3472 ( 8.2%)	3305 ( 6.0%)	< 0.001
40–49	6904 (16.3%)	7120 (12.9%)	
50–59	12,092 (28.5%)	11,606 (21.0%)	
60–69	11,781 (27.8%)	17,239 (31.2%)	
70–79	6885 (16.2%)	14,218 (25.7%)	
≥ 80	1254 ( 3.0%)	1741 ( 3.2%)	
Sex			< 0.001
Male	21,601 (51.0%)	33,530 (60.7%)	
Female	20,787 (49.0%)	21,699 (39.3%)	
Comorbidities			
Hypertension	18,477 (43.6%)	26,844 (48.6%)	< 0.001
Diabetes	8076 (19.1%)	11,549 (20.9%)	< 0.001
Dyslipidemia	14,906 (35.2%)	20,487 (37.1%)	< 0.001
CCI	2.0 ± 2.0	2.1 ± 2.0	< 0.001
0	10,112 (23.9%)	11,975 (21.7%)	< 0.001
1	10,677 (25.2%)	13,412 (24.3%)	
2	7984 (18.8%)	10,772 (19.5%)	
≥ 3	13,615 (32.1%)	19,070 (34.5%)	
BMI (kg/m^2^)	24.0 ± 3.2	24.1 ± 3.0	0.142
Drink			< 0.001
Non	26,449 (62.4%)	32,148 (58.2%)	
Mild	9514 (22.4%)	14,434 (26.1%)	
Moderate	3594 ( 8.5%)	5214 ( 9.4%)	
Severe	2831 ( 6.7%)	3433 ( 6.2%)	
Smoke			< 0.001
Never smoker	27,532 (65.0%)	33,515 (60.7%)	
Ex-smoker	7168 (16.9%)	13,626 (24.7%)	
Current smoker	7688 (18.1%)	8088 (14.6%)	
Low income			< 0.001
The bottom 20% or below	35,798 (84.5%)	47,878 (86.7%)	
Others	6590 (15.5%)	7351 (13.3%)	
Depression	11,452 (27.0%)	14,373 (26.0%)	0.001

Data are expressed as the mean ± the standard deviation or as n (%). The P-value is derived from the student t-test and the Chi-square test. PA, physical activity; BMI, body mass index; CCI. Charlson comorbidity index; OAG. open angle glaucoma. Patients with higher post-diagnosis PA levels were generally older, more likely male, and had a lower prevalence of depression than those with low PA levels.

Table 3 summarizes the baseline characteristics of the four groups categorized by changes in PA levels pre- and post-OAG diagnosis. All variables showed significant differences among groups (*p* < 0.001). The prevalence of depression varied depending on PA patterns, with the highest proportion observed in individuals who had high PA levels pre-diagnosis that were reduced post-diagnosis (High-Low level group, 28.4%). In contrast, those who maintained high PA levels pre- and post-glaucoma diagnosis exhibited the lowest prevalence of depression (High-High level group, 26.0%). The post-hoc test results (Supplementary Table 1) showed that the proportion of patients with depression differed significantly between the High-Low and High-High levels groups (*p* < 0.001). In contrast, no significant difference was observed in the proportion of patients with depression between the Low-High and High-High levels groups (*p* = 0.746).

**Table 3:** Baseline characteristics of open angle glaucoma patients stratified by changes in physical activity levels before and after diagnosis.

	Low - Low level	Low - High level	High - Low level	High - High level	*P*-value
	(*N* = 26575)	(*N* = 17257)	(*N* = 15813)	(*N* = 37972)	
Age (year, N)	56.7 ± 12.2	59.2 ± 12.6	60.0 ± 12.5	62.1 ± 12.2	< 0.001
< 40	2428 ( 9.1%)	1289 ( 7.5%)	1044 ( 6.6%)	2016 ( 5.3%)	< 0.001
40–49	4789 (18.0%)	2600 (15.1%)	2115 (13.4%)	4520 (11.9%)	
50–59	8089 (30.4%)	4351 (25.2%)	4003 (25.3%)	7255 (19.1%)	
60–69	7153 (26.9%)	4988 (28.9%)	4628 (29.3%)	12,251 (32.3%)	
70–79	3421 (12.9%)	3496 (20.3%)	3464 (21.9%)	10,722 (28.2%)	
≥ 80	695 ( 2.6%)	533 ( 3.1%)	559 ( 3.5%)	1208 ( 3.2%)	
Sex					< 0.001
Male	13,129 (49.4%)	9353 (54.2%)	8472 (53.6%)	24,177 (63.7%)	
Female	13,446 (50.6%)	7904 (45.8%)	7341 (46.4%)	13,795 (36.3%)	
Comorbidities					
Hypertension	11,112 (41.8%)	7875 (45.6%)	7365 (46.6%)	18,969 (50.0%)	< 0.001
Diabetes	4847 (18.2%)	3368 (19.5%)	3229 (20.4%)	8181 (21.5%)	< 0.001
Dyslipidemia	9168 (34.5%)	6204 (36.0%)	5738 (36.3%)	14,283 (37.6%)	< 0.001
CCI	2.0 ± 2.0	2.1 ± 2.0	2.1 ± 2.0	2.2 ± 2.0	< 0.001
0	6622 (24.9%)	3866 (22.4%)	3490 (22.1%)	8109 (21.4%)	< 0.001
1	6746 (25.4%)	4320 (25.0%)	3931 (24.9%)	9092 (23.9%)	
2	4975 (18.7%)	3307 (19.2%)	3009 (19.0%)	7465 (19.7%)	
≥ 3	8232 (31.0%)	5764 (33.4%)	5383 (34.0%)	13,306 (35.0%)	
BMI (kg/m^2^)	24.0 ± 3.3	24.1 ± 3.1	24.1 ± 3.1	24.1 ± 2.9	< 0.001
Drinking					< 0.001
Non	16,848 (63.4%)	10,955 (63.5%)	9601 (60.7%)	21,193 (55.8%)	
Mild	5709 (21.5%)	3823 (22.2%)	3805 (24.1%)	10,611 (27.9%)	
Moderate	2249 ( 8.5%)	1434 ( 8.3%)	1345 ( 8.5%)	3780 (10.0%)	
Severe	1769 ( 6.7%)	1045 ( 6.1%)	1062 ( 6.7%)	2388 ( 6.3%)	
Smoking					< 0.001
Never smoker	17,473 (65.7%)	11,256 (65.2%)	10,059 (63.6%)	22,259 (58.6%)	
Ex-smoker	4019 (15.1%)	3161 (18.3%)	3149 (19.9%)	10,465 (27.6%)	
Current smoker	5083 (19.1%)	2840 (16.5%)	2605 (16.5%)	5248 (13.8%)	
Low income					< 0.001
The bottom 20% or below	4320 (16.3%)	2534 (14.7%)	2270 (14.4%)	4817 (12.7%)	
Others	22,255 (83.7%)	14,723 (85.3%)	13,543 (85.6%)	33,155 (87.3%)	
Depression	6962 (26.2%)	4507 (26.1%)	4490 (28.4%)	9866 (26.0%)	< 0.001

Data are expressed as the mean ± the standard deviation or as n (%). The P-value is derived from the analysis of variance and the Chi-square test. Data are expressed as the mean ± the standard deviation or as n (%). The P-value is derived from the student t-test. BMI, body mass index, and CCI. Charlson comorbidity index. Depression prevalence was highest in the High–Low PA group and lowest in the High–High PA group, suggesting that maintaining high PA may be protective.

### Post-diagnosis PA and depression risk

**Fig. 1:**
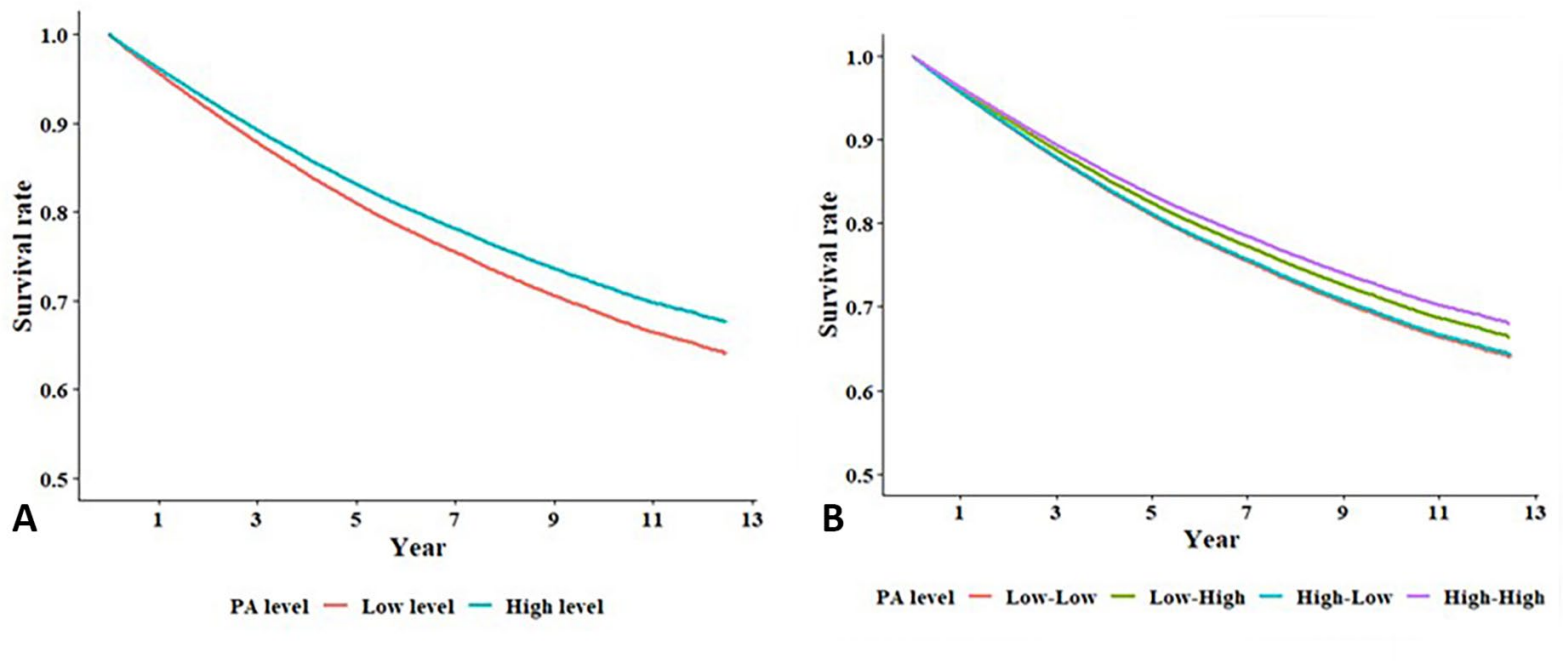
Kaplan–Meier survival curves for depression-free survival among patients with open angle glaucoma (OAG), stratified by physical activity (PA). (**A**) Depression-free survival according to PA level after OAG diagnosis. Patients were categorized into two groups based on PA level (low vs. high) (**B**) Depression-free survival according to changes in PA levels before and after OAG diagnosis. Patients were categorized into four groups: consistently low PA, consistently high PA, increased PA (low to high), and decreased PA (high to low). Depression-free survival was highest in the consistently high PA group, intermediate in the increased PA group, and lowest in the decreased PA group, highlighting the importance of maintaining PA after diagnosis.

The association between PA levels post-OAG diagnosis and risk of developing depression was examined, with individuals maintaining low post-diagnosis PA levels serving as the reference group (Supplementary Table 2, Fig. 1A). In the unadjusted model, those with high post-diagnosis PA levels demonstrated a lower hazard ratio (HR) for depression (HR = 0.954, 95% confidence interval (CI): 0.931–0.977, *p* < 0.001). After adjusting for age and sex (Model 1), the HR was further reduced to 0.866 (95% CI 0.845–0.888, *p* < 0.001). In the fully adjusted model (Model 2), which accounted for all baseline characteristics, high post-diagnosis PA levels remained significantly associated with a reduced risk of depression (HR = 0.877, 95% CI 0.856–0.900, *p* < 0.001).

### Effects of physical activity level changes on depression risk

Further analysis was conducted to assess the impact of changes in PA levels pre- and post-OAG diagnosis on depression risk, using individuals with consistently low PA levels pre- and post-diagnosis as the reference group (Table 4; Fig. 1B). In the fully adjusted model (Model 2), those who increased their PA levels from low-to-high post-diagnosis exhibited a significantly low risk of depression (HR = 0.912, 95% CI 0.879–0.947, *p* < 0.001). The lowest depression risk was observed among individuals who maintained high PA levels pre- and post-diagnosis (HR = 0.855, 95% CI 0.829–0.883, *p* < 0.001). Conversely, those who had high PA levels pre-diagnosis but reduced activity post-diagnosis did not exhibit a significant difference in depression risk compared to that of the reference group (HR = 0.989, 95% CI 0.952–1.027, *p* = 0.564).

**Table 4:** Hazard ratios for depression based on changes in physical activity levels before and after open angle glaucoma diagnosis.

	Unadjusted			Model 1			Model 2		
	HR	95% CI	*P*-value	HR	95% CI	*P*-value	HR	95% CI	*P*-value
Low–Low level	1	–	–	1	–	–	1	–	–
Low–High level	0.993	(0.957, 1.031)	0.715	0.908	(0.874, 0.943)	< 0.001	0.912	(0.879, 0.947)	< 0.001
High–Low level	1.104	(1.063, 1.146)	< 0.001	0.981	(0.944, 1.018)	0.307	0.989	(0.952, 1.027)	0.564
High–High level	0.989	(0.959, 1.02)	0.481	0.837	(0.811, 0.864)	< 0.001	0.855	(0.829, 0.883)	< 0.001

Model 1 is adjusted for age and sex. Model 2 is adjusted Model 2 was adjusted for all variables used to describe the baseline characteristics.HR, hazard ratio; CI, confidence interval; PA, physical activity. In the fully adjusted model, sustained high PA (High–High) and increased PA (Low–High) were associated with significantly reduced depression risk, whereas decreased PA (High–Low) showed no protective effect.

## Discussion

We evaluated the impact of PA on the risk of depression in patients with OAG. Our findings demonstrated that maintaining high PA levels after an OAG diagnosis was associated with a significantly lower risk of developing depression (HR = 0.877). Furthermore, patients who sustained high PA levels pre- and post-diagnosis or increased their PA levels post-diagnosis exhibited a reduced risk of depression, with HRs of 0.855 and 0.912, respectively. These findings suggest that maintaining high PA levels or enhancing PA levels may mitigate depression risk among patients with OAG, reinforcing the need to integrate PA as a component of comprehensive glaucoma management.

Progressive visual impairment is often associated with uncertainty and vulnerability, ultimately compromising an individual’s perceived autonomy^21^. The pervasive fear of complete blindness can engender significant psychological distress, influencing various aspects of daily life^22^. Numerous studies have demonstrated an increased risk of depression in patients with ophthalmic diseases that threaten vision. One such study reported that one-third of the individuals affected by visual impairment and debilitating ocular conditions exhibit mild depressive symptoms^23^. Hwang et al.^24^ reported that individuals with visual disability secondary to age-related macular degeneration had an HR of 1.15 for developing depression, with the risk further increasing to 1.23 when the visual disability was severe. Kim et al.^25^ highlighted a 1.19-fold increased risk of depressive disorders among patients with retinitis pigmentosa. We found that patients with OAG had a 1.13-fold higher risk of developing depression than the control group of individuals without OAG (Supplementary Table 1). These findings align with prior research and emphasize the mental health burden associated with chronic visual impairments. Several factors have been associated with an increased risk of depression in patients with glaucoma. These include more rapid progression of visual field loss, advanced disease severity, female sex, and, in some cases, younger age, as suggested by some studies^9,10,26^. The presence of comorbid conditions, including hypertension and diabetes mellitus, has also been linked to depressive symptoms^9^. Notably, improving patient education regarding glaucoma and its progression may help alleviate psychological distress, underscoring the importance of informed disease management^26^.

Beyond the psychological impact of vision loss, glaucoma and depression may also share biological mechanisms. Both conditions have been associated with autonomic dysfunction,^27,28^ neuroinflammation,^29–34^ and dysregulation of neurotrophic factors such as brain-derived neurotrophic factor (BDNF), leading to retinal ganglion cell (RGC) degeneration^35,36^. These common pathways may help explain why physical activity, which is known to improve autonomic balance, reduce inflammation, and enhance neurotrophic support, could mitigate depression risk in patients with glaucoma.

Depression in glaucoma patients is not merely a comorbid condition—it can undermine treatment adherence. Research indicates that depression is linked to poor adherence to medication across various chronic diseases^37–39^. Spencer et al.^40^ identified that, in addition to difficulty applying eye drops, a past or current diagnosis of depression is a significant factor contributing to reduced adherence to glaucoma treatment. Therefore, assessing the presence of depression in patients with glaucoma and considering appropriate management strategies is an important aspect in the treatment of glaucoma.

The beneficial effects of PA on both physical and mental health are well-documented. Lee et al.^41^ demonstrated that high PA levels after colorectal cancer diagnosis were associated with reduced all-cause and colorectal cancer-specific mortality. Similarly, Kim et al.^42^ reported that regular PA reduced the risk of progression from mild cognitive impairment to dementia. Several studies have demonstrated that elevated levels of PA are associated with a decreased risk of developing depression^13,43,44^. Beyond its preventive role, PA is as an effective intervention in the treatment of depression. A systematic review and network meta-analysis concluded that exercise is an effective treatment for depression, with activities such as walking, jogging, yoga, and strength training showing notable efficacy^45^. The clinical significance of PA extends beyond depression itself, as it is instrumental in managing various pathological conditions closely linked to depression, including glaucoma. Huang et al.^46^ demonstrated that glaucoma adversely impacts psychological function and daily PA, with reduced social function and mental health observed in patients with advanced-stage glaucoma. The study suggests that encouraging PA in these patients potentially improved their psychological function and quality of life. PA induces a range of positive effects, including neuroprotective benefits that contribute to overall brain health. One of the key mechanisms underlying these effects is the upregulation of neurotrophic factors, particularly BDNF, which plays a crucial role in neuronal survival, synaptic plasticity, and cognitive function^47–49^. Additionally, exercise improves mitochondrial function, which is essential for maintaining cellular energy production and reducing oxidative stress^50–52^. These mechanisms collectively highlight the potential of PA as a non-pharmacological intervention to preserve neurological function and reduce the risk of neurodegenerative diseases. Although the interrelationship between glaucoma, depression, and PA is multifaceted, PA is a modifiable factor with the potential to positively impact both ocular health and mental well-being. Nevertheless, the feasibility of promoting PA in elderly glaucoma patients with visual impairment requires careful consideration. Our study did not evaluate the specific types or intensities of PA that are most suitable for this population. Given that these patients may face unique barriers to engaging in physical activity, future research is warranted to identify safe and achievable PA strategies tailored to their clinical circumstances. In addition, the null result observed in the High–Low PA group suggests that the protective benefit of PA is reversible once activity declines, emphasizing the importance of maintaining PA levels in glaucoma patients. Reduced PA may also serve as a potential marker of disease activity, systemic health deterioration, or emerging depression, indicating that PA monitoring itself could be a clinically meaningful indicator in glaucoma management.

This study has limitations. First, disease identification was based on diagnostic codes, which may underestimate depression by missing uncoded cases. Cultural factors in Korea, such as stigma toward psychiatric diagnoses, may further contribute to under-diagnosis. Antidepressant prescription data were not incorporated, but the use of depression codes (F32, F33) in Korea does not necessarily require concurrent medication. Thus, our approach is consistent with prior KNHIS-based studies and remains valid for identifying depression cases. In addition, clinical indicators of glaucoma severity (e.g., visual field indices or optic nerve status) were not available in the KNHIS database, which may weaken the validity of our finding since disease severity could mediate both PA and depression risk. Nevertheless, this limitation is inherent to claims-based data, and the use of a large customized KNHIS dataset still provides robust and clinically meaningful evidence. Second, our study included only individuals who had undergone health examinations, which may introduce selection bias. Third, the PA data were self-reported and may therefore be subject to recall bias and social desirability bias. Such potential misclassification of PA levels is likely to be nondifferential, which could bias the hazard ratios toward the null. As a result, the observed associations in our study may have underestimated the true protective effect of physical activity against depression risk. In addition, PA was analyzed using dichotomized categories rather than continuous scores, following the methodology used in previous large-scale epidemiologic studies. While this approach enhances comparability and clinical interpretability, it precluded a more detailed assessment of dose–response relationships, which should be addressed in future research. Fourth, the study cohort predominantly comprised individuals of Korean ethnicity, limiting the applicability of our findings to other populations. Finally, despite the longitudinal nature of our dataset, this study remains retrospective in design. Nonetheless, our study has several notable strengths. A major advantage is the utilization of a large, nationwide cohort with customized data from the KNHIS, enabling comprehensive analysis with enhanced generalizability. Additionally, the stratification of patients based on changes in PA levels pre- and post-OAG diagnosis offers unique insights into the dynamic association between PA and depression risk.

In conclusion, our study suggests that higher PA levels are associated with a modest but meaningful reduction in depression risk among patients with OAG. Maintaining or increasing PA levels may help mitigate the risk of depression, underscoring the value of encouraging PA as part of comprehensive glaucoma care.

## Methods

The Institutional Review Board of Asan Medical Center and University of Ulsan College of Medicine approved a waiver to review the data for this study (2024 − 0116). This study was conducted according to the ethical principles outlined in the Declaration of Helsinki, and the requirement for informed consent was waived due to the use of anonymized and de-identified information.

This nationwide, population-based, retrospective cohort study used a customized database of the KNHIS between 2009 and 2023. The detailed characteristics of the KNHIS data have been described in several studies and are briefly summarized here^53,54^. The health insurance system of South Korea offers comprehensive data on age, sex, general health status, medical examination records obtained through the health screening program, and disease diagnoses classified using the Korean Standard Classification of Disease (KCD) codes, similar to those of the International Classification of Disease. Additionally, it includes information on prescribed medications, medical procedures including diagnostic tests and hospital visit histories, facilitated by the electronic exchange of cost-related healthcare data between the KNHIS and medical providers through the Korean Electronic Data Interchange medical procedure codes. The KNHIS provides nationwide health examination to the general Korean population every 2 years to facilitate early detection and prevent diseases. The information collected through these screening programs includes answers to standardized questionnaires, physical examinations, and laboratory test outcomes.

### Definition of database and patients

The flowchart depicting the study population is presented in Supplementary Fig. 1. This study utilized a customized database specifically comprising records related to patients with OAG between 2011 and 2015 along with KNHIS data of patients between 2009 and 2023. However, due to changes in health examination surgery items in 2009, records prior to 2009 were excluded, and patients who had already been diagnosed with OAG between 2009 and 2010 were excluded. The inclusion criteria required patients to have at least one health examination record during this period. Patients were followed to the earliest occurrence of newly diagnosed depression, death, or end of the study period (December 31, 2023). We established a control group of participants without a history of OAG, age- and sex-matched to the patient group at a 1:2 ratio. Data from all patients diagnosed with OAG between January 1, 2011 and December 31, 2015 were analyzed (*n* = 167,725). Among these, we included only those who had undergone a health examination within 2 years following their OAG diagnosis (*n* = 131195). Patients who had been diagnosed with depression prior to the index date, defined as the date of their second health examination (*n* = 30358), and those with missing data (*n* = 3220) were excluded. Finally, 97,617 individuals were included in this study.

OAG was defined based on the fulfillment of the following criteria: (1) a diagnosis of OAG confirmed by the KCD code (H401), (2) completion of at least one visual field test, and (3) receipt of a prescription for anti-glaucoma medications^55,56^. Individuals with diagnostic codes F32 and F33 during follow-up were considered to have a newly diagnosed depression.

### PA measurements

PA was assessed during the health examination using questions related to the weekly frequency of vigorous and moderate intensity PA as well as walking. Based on a previous study,^41^ we classified PA into two categories: low- and high-level PA. Individuals with a total score of ≤ 2 were categorized into the low-level PA group, whereas those with a score ≥ 3 were categorized into the high-level PA group. The PA score for individuals < 65 years was calculated by adding the score for moderate exercise to twice the score for vigorous exercise. For individuals aged ≥ 65, the scores for moderate and walking were added to twice the score for vigorous exercise.

We investigated the impact of changes in PA on the incidence of depression by classifying patients with OAG into four groups based on PA levels within 2 years pre- and post-OAG diagnosis. The four groups were defined as follows: patients with OAG maintained either low- or high-level PA pre- and post-OAG diagnosis, those whose PA level increased from low-level before the diagnosis to high-level after the diagnosis, and those whose PA level decreased from high-level before the diagnosis to low-level after the diagnosis.

### Other variables: comorbidities and sociodemographic factors

Comorbid conditions, including hypertension (HTN), diabetes mellitus (DM), and dyslipidemia, were identified based on health examination measurements, diagnostic codes, and prescription codes. HTN was defined as systolic blood pressure ≥ 140 mmHg, diastolic blood pressure ≥ 90 mmHg, or the presence of the KCD code I10-14 and I15. DM was defined as fasting blood glucose ≥ 126 mg/dl or presence of KCD codes E10-E14. Dyslipidemia was defined as total cholesterol ≥ 240 mg/dl or the presence of KCD code E78. The CCI was utilized as a covariate to account for and correspond to the overall general health status between groups^57^. This index represents a weighted score derived from the presence of 17 systemic diseases, with higher scores indicating a greater burden of systemic comorbidities. BMI, alcohol consumption, smoking status, and income level were defined using the questionnaire results. BMI was determined by dividing the weight in kilograms by the square of height in meters (kg/m^2^). Alcohol consumption was classified based on the weekly intake of alcohol: mild drinking, < 105 g/week; heavy drinking, ≥ 210 g/week; moderate drinking, 105–210 g/week. Patients were classified into non-smokers, former smokers, and never smokers based on the smoking status. Low household income level was defined as being within the lowest 20% of income distribution.

### Statistical analysis

Differences in the distribution of baseline characteristics were assessed using Student t-test or analysis of variance for continuous variables, and the chi-square test for categorical variables. Bonferroni correction was used as the post-hoc test, and the Cox proportional hazards model was employed to estimate the HR and 95% CI for newly diagnosed depression. The proportional hazards assumption for all Cox regression models was evaluated using Schoenfeld residuals, and no significant violations were detected. In addition, tests of the proportional hazards assumption using Schoenfeld residuals indicated no significant violations, supporting the validity of the Cox regression models. Multivariable analyses were performed using two models, each based on different sets of adjustment variables. Model 1 was adjusted for age and sex, whereas Model 2 was adjusted for all variables used to describe the baseline characteristics. A two-sided p-value of < 0.05 was considered statistically significant. All statistical analyses were performed using SAS version 9.4 (SAS Institute Inc.) and R Statistical Software version 4.0.3 (Foundation for Statistical Computing).

## Supplementary Information

Below is the link to the electronic supplementary material.

Supplementary Material 1

Supplementary Material 2

## Data Availability

The datasets used and analyzed during the current study are not publicly available due to national data protection regulations governing the Korean National Health Insurance Service (KNHIS) database. However, access to the data may be granted by the NHIS for researchers who meet the required conditions for data use. Requests for data access should be directed to the NHIS (https://nhiss.nhis.or.kr/).

## References

[1] Klein, B. E., Moss, S. E., Klein, R., Lee, K. E. & Cruickshanks, K. J. Associations of visual function with physical outcomes and limitations 5 years later in an older population: The beaver dam eye study. *Ophthalmology* **110**, 644–650 (2003). 12689880 10.1016/S0161-6420(02)01935-8

[2] Bobo, W. V. et al. Association of depression and anxiety with the accumulation of chronic conditions. *JAMA Netw. Open.* **5**, e229817 (2022). 35499825 10.1001/jamanetworkopen.2022.9817 PMC9062691

[3] Guo, D., Wang, C. & Liu, X. Association of chronic diseases with depression in the united States, NHANES 2007–2018. *Psychol. Health Med.* **29**, 1077–1090 (2024). 37990352 10.1080/13548506.2023.2277153

[4] Scott, A. J., Correa, A. B., Bisby, M. A. & Dear, B. F. Depression and anxiety trajectories in chronic disease: A systematic review and meta-analysis. *Psychother. Psychosom.* **92**, 227–242 (2023). 37607505 10.1159/000533263

[5] Ali, S., Stone, M. A., Peters, J. L., Davies, M. J. & Khunti, K. The prevalence of co-morbid depression in adults with type 2 diabetes: A systematic review and meta-analysis. *Diabet. Med.* **23**, 1165–1173 (2006). 17054590 10.1111/j.1464-5491.2006.01943.x

[6] Kwapong, Y. A. et al. Association of depression and poor mental health with cardiovascular disease and suboptimal cardiovascular health among young adults in the United States. *J. Am. Heart Assoc.* **12**, e028332 (2023). 36688365 10.1161/JAHA.122.028332 PMC9973664

[7] Paile-Hyvärinen, M. et al. Depression and its association with diabetes, cardiovascular disease, and birth weight. *Ann. Med.* **39**, 634–640 (2007). 17852029 10.1080/07853890701545722

[8] Chen, Y. Y. et al. The association between glaucoma and risk of depression: A nationwide population-based cohort study. *BMC Ophthalmol.* **18**, 146 (2018). 29929494 10.1186/s12886-018-0811-5 PMC6013853

[9] Stamatiou, M. E., Kazantzis, D., Theodossiadis, P. & Chatziralli, I. Depression in glaucoma patients: A review of the literature. *Semin Ophthalmol.* **37**, 29–35 (2022). 33822676 10.1080/08820538.2021.1903945

[10] Zhang, X. et al. The association between glaucoma, anxiety, and depression in a large population. *Am. J. Ophthalmol.* **183**, 37–41 (2017). 28760639 10.1016/j.ajo.2017.07.021

[11] Herring, M. P., Puetz, T. W., O’Connor, P. J. & Dishman, R. K. Effect of exercise training on depressive symptoms among patients with a chronic illness: a systematic review and meta-analysis of randomized controlled trials. *Arch. Intern. Med.* **172**, 101–111 (2012). 22271118 10.1001/archinternmed.2011.696

[12] Lee, J. M. & Ryan, E. J. The relationship between the frequency and duration of physical activity and depression in older adults with multiple chronic diseases. *J Clin. Med* **11** (2022). 10.3390/jcm11216355 PMC9657565 36362583

[13] Pearce, M. et al. Association between physical activity and risk of depression: A systematic review and Meta-analysis. *JAMA Psychiatry*. **79**, 550–559 (2022). 35416941 10.1001/jamapsychiatry.2022.0609 PMC9008579

[14] Kandola, A., Ashdown-Franks, G., Hendrikse, J., Sabiston, C. M. & Stubbs, B. Physical activity and depression: Towards understanding the antidepressant mechanisms of physical activity. *Neurosci. Biobehav Rev.* **107**, 525–539 (2019). 31586447 10.1016/j.neubiorev.2019.09.040

[15] Seo, D. Y., Heo, J. W., Ko, J. R. & Kwak, H. B. Exercise and neuroinflammation in health and disease. *Int. Neurourol. J.* **23**, S82–92 (2019). 31795607 10.5213/inj.1938214.107 PMC6905205

[16] Cho, D. H., Jae, S. Y., Kunutsor, S., Choi, J. & Gwon, J. G. Longitudinal increase in physical activity and adverse cardiovascular outcomes following the diagnosis of acute coronary syndrome. *Br. J. Sports Med.* **59**, 774–782 (2025). 39814538 10.1136/bjsports-2024-108923

[17] Choi, J. et al. Impact of pre- and post-diagnosis physical activity on the mortality of patients with cancer: Results from the health examinees-G study in Korea. *Cancer Med.* **12**, 16591–16603 (2023). 37317668 10.1002/cam4.6253 PMC10469756

[18] Gerber, Y. et al. Trajectories of physical activity before and after cardiovascular disease events in CARDIA participants. *JAMA Cardiol.* **10**, 949–953 (2025). 40699583 10.1001/jamacardio.2025.2282 PMC12287934

[19] Lei, Y. Y. et al. Longitudinal changes in sports activity from pre-diagnosis to first five years post-diagnosis: A prospective Chinese breast cancer cohort study. *BMC Cancer*. **20**, 1013 (2020). 33076863 10.1186/s12885-020-07517-6 PMC7574482

[20] Preiss, D. et al. Change in levels of physical activity after diagnosis of type 2 diabetes: An observational analysis from the NAVIGATOR study. *Diabetes Obes. Metab.* **16**, 1265–1268 (2014). 24861892 10.1111/dom.12320

[21] Demmin, D. L. & Silverstein, S. M. Visual impairment and mental health: Unmet needs and treatment options. *Clin. Ophthalmol.* **14**, 4229–4251 (2020). 33299297 10.2147/OPTH.S258783 PMC7721280

[22] Sabel, B. A., Wang, J., Cárdenas-Morales, L., Faiq, M. & Heim, C. Mental stress as consequence and cause of vision loss: The dawn of psychosomatic ophthalmology for preventive and personalized medicine. *Epma j.* **9**, 133–160 (2018). 29896314 10.1007/s13167-018-0136-8 PMC5972137

[23] Rees, G. et al. Vision-specific distress and depressive symptoms in people with vision impairment. *Invest. Ophthalmol. Vis. Sci.* **51**, 2891–2896 (2010). 20164466 10.1167/iovs.09-5080

[24] Hwang, S. et al. Impact of Age-Related macular degeneration and related visual disability on the risk of depression: A nationwide cohort study. *Ophthalmology* **130**, 615–623 (2023). 36717001 10.1016/j.ophtha.2023.01.014

[25] Kim, H. R. et al. Incidence and risk of depressive disorder in patients with retinitis pigmentosa. *JAMA Ophthalmol.* **142**, 997–1004 (2024). 39298178 10.1001/jamaophthalmol.2024.3641 PMC11413759

[26] Lim, N. C., Fan, C. H., Yong, M. K., Wong, E. P. & Yip, L. W. Assessment of Depression, Anxiety, and quality of life in Singaporean patients with glaucoma. *J. Glaucoma*. **25**, 605–612 (2016). 26950574 10.1097/IJG.0000000000000393

[27] Wierzbowska, J., Wierzbowski, R., Stankiewicz, A., Siesky, B. & Harris, A. Cardiac autonomic dysfunction in patients with normal tension glaucoma: 24-h heart rate and blood pressure variability analysis. *Br. J. Ophthalmol.* **96**, 624–628 (2012). 22399689 10.1136/bjophthalmol-2011-300945

[28] Sgoifo, A., Carnevali, L., Alfonso Mde, L. & Amore, M. Autonomic dysfunction and heart rate variability in depression. *Stress* **18**, 343–352 (2015). 26004818 10.3109/10253890.2015.1045868

[29] Križaj, D. et al. From mechanosensitivity to inflammatory responses: new players in the pathology of glaucoma. *Curr. Eye Res.* **39**, 105–119 (2014). 24144321 10.3109/02713683.2013.836541 PMC3946931

[30] Yuan, L. & Neufeld, A. H. Tumor necrosis factor-alpha: a potentially neurodestructive cytokine produced by glia in the human glaucomatous optic nerve head. *Glia* **32**, 42–50 (2000). 10975909

[31] Tezel, G., Li, L. Y., Patil, R. V. & Wax, M. B. TNF-alpha and TNF-alpha receptor-1 in the retina of normal and glaucomatous eyes. *Invest. Ophthalmol. Vis. Sci.* **42**, 1787–1794 (2001). 11431443

[32] Malhi, G. S. & Mann, J. J. *Depress. Lancet* **392**, 2299–2312 (2018). 10.1016/S0140-6736(18)31948-2 30396512

[33] Tezel, G. TNF-alpha signaling in glaucomatous neurodegeneration. *Prog Brain Res.* **173**, 409–421 (2008). 18929124 10.1016/S0079-6123(08)01128-X PMC3150483

[34] Wei, X., Cho, K. S., Thee, E. F., Jager, M. J. & Chen, D. F. Neuroinflammation and microglia in glaucoma: time for a paradigm shift. *J. Neurosci. Res.* **97**, 70–76 (2019). 29775216 10.1002/jnr.24256 PMC6239948

[35] Quigley, H. A. et al. Retrograde axonal transport of BDNF in retinal ganglion cells is blocked by acute IOP elevation in rats. *Invest. Ophthalmol. Vis. Sci.* **41**, 3460–3466 (2000). 11006239

[36] Liu, W. et al. The Role of Neural Plasticity in Depression: From Hippocampus to Prefrontal Cortex. *Neural Plast* 6871089 (2017). (2017). 10.1155/2017/6871089 PMC5299163 28246558

[37] Grenard, J. L. et al. Depression and medication adherence in the treatment of chronic diseases in the united states: a meta-analysis. *J. Gen. Intern. Med.* **26**, 1175–1182 (2011). 21533823 10.1007/s11606-011-1704-y PMC3181287

[38] Qian, J. et al. Association between depression and maintenance medication adherence among medicare beneficiaries with chronic obstructive pulmonary disease. *Int. J. Geriatr. Psychiatry*. **29**, 49–57 (2014). 23606418 10.1002/gps.3968 PMC3797159

[39] Alomar, A. O. et al. Relationship between depression and medication adherence among chronic disease patients in the middle East. *Cureus* **16**, e69418 (2024). 39403637 10.7759/cureus.69418 PMC11473096

[40] Spencer, S. K. R. et al. Factors affecting adherence to topical glaucoma therapy: A quantitative and qualitative pilot study analysis in Sydney, Australia. *Ophthalmol. Glaucoma*. **2**, 86–93 (2019). 32672609 10.1016/j.ogla.2019.01.006

[41] Lee, M., Lee, Y., Jang, D. & Shin, A. Physical activity after colorectal cancer diagnosis and mortality in a nationwide retrospective cohort study. *Cancers (Basel)* 13 (2021). 10.3390/cancers13194804 PMC8508146 34638290

[42] Kim, Y. J. et al. Association between physical activity and conversion from mild cognitive impairment to dementia. *Alzheimers Res. Ther.* **12**, 136 (2020). 33176851 10.1186/s13195-020-00707-1 PMC7661208

[43] Mammen, G. & Faulkner, G. Physical activity and the prevention of depression: a systematic review of prospective studies. *Am. J. Prev. Med.* **45**, 649–657 (2013). 24139780 10.1016/j.amepre.2013.08.001

[44] Schuch, F. B. et al. Physical activity and incident depression: A Meta-Analysis of prospective cohort studies. *Am. J. Psychiatry*. **175**, 631–648 (2018). 29690792 10.1176/appi.ajp.2018.17111194

[45] Noetel, M. et al. Effect of exercise for depression: systematic review and network meta-analysis of randomised controlled trials. *Bmj* **384**, e075847 (2024). 38355154 10.1136/bmj-2023-075847 PMC10870815

[46] Huang, W., Gao, K., Liu, Y., Liang, M. & Zhang, X. The Adverse Impact of Glaucoma on Psychological Function and Daily Physical Activity. *J Ophthalmol* 9606420 (2020). (2020). 10.1155/2020/9606420 PMC7191398 32377425

[47] Lu, B., Nagappan, G. & Lu, Y. BDNF and synaptic plasticity, cognitive function, and dysfunction. *Handb. Exp. Pharmacol.* **220**, 223–250 (2014). 24668475 10.1007/978-3-642-45106-5_9

[48] Jaberi, S. & Fahnestock, M. Mechanisms of the Beneficial Effects of Exercise on Brain-Derived Neurotrophic Factor Expression in Alzheimer’s Disease. *Biomolecules* 13 (2023). 10.3390/biom13111577 PMC10669442 38002258

[49] Ceylan, H., Silva, A. F. & Ramirez-Campillo, R. & Murawska-Ciałowicz, E. Exploring the effect of acute and regular physical exercise on Circulating Brain-Derived neurotrophic factor levels in individuals with obesity: A comprehensive systematic review and Meta-Analysis. *Biology (Basel)* 13 (2024). 10.3390/biology13050323 PMC11117522 38785805

[50] Demine, S., Renard, P. & Arnould, T. Mitochondrial uncoupling: A key controller of biological processes in physiology and diseases. *Cells* **8** (2019). 10.3390/cells8080795 PMC6721602 31366145

[51] Angulo, J., El Assar, M. & Álvarez-Bustos, A. Rodríguez-Mañas, L. Physical activity and exercise: strategies to manage frailty. *Redox Biol.* **35**, 101513 (2020). 32234291 10.1016/j.redox.2020.101513 PMC7284931

[52] O’Reilly, C. L., Miller, B. F. & Lewis, T. L. Jr. Exercise and mitochondrial remodeling to prevent age-related neurodegeneration. *J. Appl. Physiol. (1985)*. **134**, 181–189 (2023). 36519568 10.1152/japplphysiol.00611.2022 PMC9829476

[53] Kim, M. K., Han, K. & Lee, S. H. Current trends of big data research using the Korean National health information database. *Diabetes Metab. J.* **46**, 552–563 (2022). 35929173 10.4093/dmj.2022.0193 PMC9353560

[54] Kyoung, D. S. & Kim, H. S. Assessment (HIRA) Database for Research. *J. Lipid Atheroscler*. **11**, 103–110 (2022). Understanding and Utilizing Claim Data from the Korean National Health Insurance Service (NHIS) and Health Insurance Review. 10.12997/jla.2022.11.2.103 PMC9133780 35656154

[55] Lee, S. Y., Lee, J. S., Kim, J. Y., Tchah, H. & Lee, H. Visit-to-visit variability in blood pressure and the risk of open-angle glaucoma in individuals without systemic hypertension: a nationwide population-based cohort study. *Front. Med. (Lausanne)*. **10**, 1300778 (2023). 38269321 10.3389/fmed.2023.1300778 PMC10805885

[56] Rim, T. H., Lee, S. Y., Kim, S. H., Kim, S. S. & Kim, C. Y. Increased incidence of open-angle glaucoma among hypertensive patients: an 11-year nationwide retrospective cohort study. *J. Hypertens.* **35**, 729–736 (2017). 28253217 10.1097/HJH.0000000000001225

[57] Charlson, M. E., Pompei, P., Ales, K. L. & MacKenzie, C. R. A new method of classifying prognostic comorbidity in longitudinal studies: development and validation. *J. Chronic Dis.* **40**, 373–383 (1987). 3558716 10.1016/0021-9681(87)90171-8

