# Supplementary figures and images for: Independent associations of physical activity and depression with open-angle glaucoma in a population-based analysis
Source: Sci Rep. 2025 Nov 21;15:44555. doi: 10.1038/s41598-025-28364-0 (PMC12739159; doi:10.1038/s41598-025-28364-0)

**Supplementary Figure 1.** Flow diagram illustrating the selection of study participants

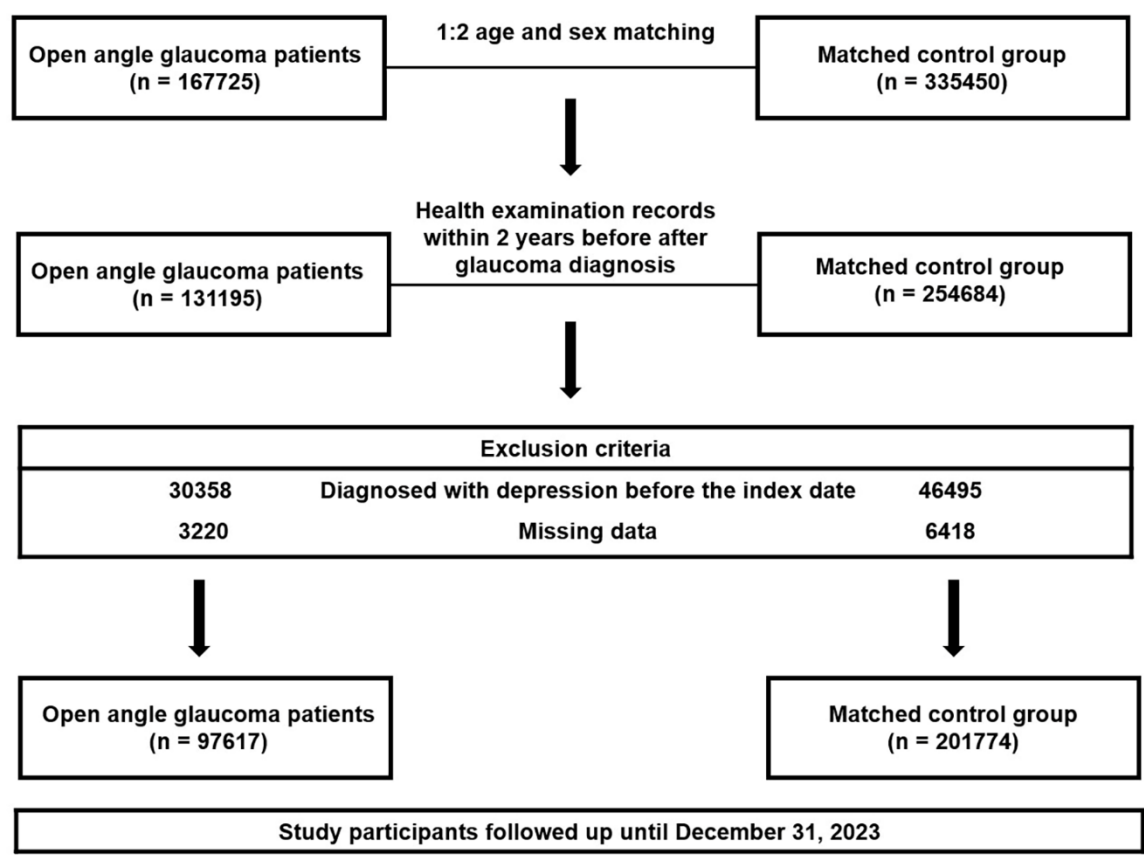

Supplement: Supplementary file 1 — Supplementary Material 1 [file 41598_2025_28364_MOESM1_ESM.pdf]
