# Supplementary material for: Independent associations of physical activity and depression with open-angle glaucoma in a population-based analysis
Source: Sci Rep. 2025 Nov 21;15:44555. doi: 10.1038/s41598-025-28364-0 (PMC12739159; doi:10.1038/s41598-025-28364-0)
Supplement: Supplementary file 2 — Supplementary Material 2 [file 41598_2025_28364_MOESM2_ESM.docx]

**Supplementary Table 1.** Post hoc test results among groups stratified by physical activity (PA) level before and after open-angle glaucoma (OAG) diagnosis. Patients were categorized into four groups based on PA changes: Low–Low (low PA both before and after OAG diagnosis), Low–High (low PA before but high PA after diagnosis), High–Low (high PA before but low PA after diagnosis), and High–High (high PA both before and after diagnosis). Bonferroni correction was used for the post hoc test. BMI, body mass index; CCI, Charlson comorbidity index.

|  | **Post hoc test P-value*** | | | | | |
| --- | --- | --- | --- | --- | --- | --- |
|  | Low-Low vs Low-High | Low-Low vs High-Low | Low-Low vs High-High | Low-High vs High-Low | Low-High vs High-High | High-Low vs High-High |
| **Age** |  |  |  |  |  |  |
|  | <0.001 | <0.001 | <0.001 | <0.001 | <0.001 | <0.001 |
| < 40 | <0.001 | <0.001 | <0.001 | <0.001 | <0.001 | <0.001 |
| 40-49 |  |  |  |  |  |  |
| 50-59 |  |  |  |  |  |  |
| 60-69 |  |  |  |  |  |  |
| 70-79 |  |  |  |  |  |  |
| ≥ 80 |  |  |  |  |  |  |
| **Sex** | <0.001 | <0.001 | <0.001 | 0.261 | <0.001 | <0.001 |
| Male |  |  |  |  |  |  |
| Female |  |  |  |  |  |  |
| **Comorbidities** |  |  |  |  |  |  |
| Hypertension | <0.001 | <0.001 | <0.001 | 0.081 | <0.001 | <0.001 |
| Diabetes | <0.001 | <0.001 | <0.001 | 0.041 | <0.001 | 0.004 |
| Dyslipidemia | 0.002 | <0.001 | <0.001 | 0.533 | <0.001 | 0.004 |
| **CCI** |  |  |  |  |  |  |
|  | <0.001 | <0.001 | <0.001 | 0.598 | <0.001 | 0.420 |
| 0 | <0.001 | <0.001 | <0.001 | 0.661 | <0.001 | 0.007 |
| 1 |  |  |  |  |  |  |
| 2 |  |  |  |  |  |  |
| ≥ 3 |  |  |  |  |  |  |
| **BMI (kg/m^2^)** | 0.046 | <0.001 | 0.013 | 0.992 | >0.999 | 0.449 |
| **Drinking** | 0.039 | <0.001 | <0.001 | <0.001 | <0.001 | <0.001 |
| Non |  |  |  |  |  |  |
| Mild |  |  |  |  |  |  |
| Moderate |  |  |  |  |  |  |
| Severe |  |  |  |  |  |  |
| **Smoking** | <0.001 | <0.001 | <0.001 | <0.001 | <0.001 | <0.001 |
| Never smoker |  |  |  |  |  |  |
| Ex-smoker |  |  |  |  |  |  |
| Current smoker |  |  |  |  |  |  |
| **Low income** | <0.001 | <0.001 | <0.001 | 0.406 | <0.001 | <0.001 |
| The bottom 20% or below |  |  |  |  |  |  |
| Others |  |  |  |  |  |  |
| **Depression** | 0.859 | <0.001 | <0.001 | <0.001 | 0.746 | <0.001 |

*Bonferroni correction was used for the post hoc test.

BMI, body mass index; CCI. Charlson comorbidity index.

**Supplementary Table 2.** Hazard ratios for depression based on post-diagnosis physical activity level in open angle glaucoma patients

|  | **Unadjusted** | | | **Model 1** | | | **Model 2** | | |
| --- | --- | --- | --- | --- | --- | --- | --- | --- | --- |
|  | **HR** | **95% CI** | **P-value** | **HR** | **95% CI** | **P-value** | **HR** | **95% CI** | **P-value** |
| **PA low level** | 1 | - | - | 1 | - | - | 1 | - | - |
| **PA high level** | 0.954 | (0.931, 0.977) | <0.001 | 0.866 | (0.845, 0.888) | <0.001 | 0.877 | (0.856, 0.900) | <0.001 |

Model 1 is adjusted for age and sex. Model 2 is adjusted Model 2 was adjusted for all variables used to describe the baseline characteristics.HR, hazard ratio; CI, confidence interval; PA, physical activity.
